# Experimental Performance Comparison of a Modular Water-Based Photovoltaic–Thermal System Under Multiple Hydraulic Operating Modes in a Tropical Climate

**DOI:** 10.3390/s26103108

**Published:** 2026-05-14

**Authors:** Carlos Roberto Coutinho, Rodrigo Fiorotti, Marcelo Eduardo Vieira Segatto, Jussara Farias Fardin, Helder Roberto de Oliveira Rocha

**Affiliations:** 1 Department of the Electrical Engineering, Federal Institute of Espírito Santo, Rod. BR-101, km 58, São Mateus 29932-540, ES, Brazil; rodrigo.fiorotti@ifes.edu.br; 2 Department of Electrical Engineering, Federal University of Espírito Santo, Av. Fernando Ferrari, 514, Vitoria 29075-910, ES, Brazil; marcelo.segatto@ufes.br (M.E.V.S.); jussara.fardin@ufes.br (J.F.F.)

**Keywords:** hybrid solar system, thermal energy recovery, forced circulation, thermosiphon, real-time monitoring, energy efficiency, tropical climate

## Abstract

In Brazil, more than 80% of households rely on electricity for water heating, representing approximately 13% of residential electricity consumption and significantly contributing to peak grid demand. As a prominent alternative for supplying household thermal energy and reducing grid stress, this study experimentally evaluates, under tropical climate conditions, the performance of a modular water-based photovoltaic–thermal (PVT) system and compares it with a conventional photovoltaic (PV) system operating simultaneously under identical environmental conditions. The PVT system, based on commercial PV modules coupled to roll-bond heat exchangers, a storage tank, and a shower outlet, was tested under three hydraulic regimes: natural thermosiphon, closed-loop, and Forced circulation. A dedicated ESP32-based data acquisition system, integrated with a cloud platform, continuously monitors electrical, thermal, and meteorological variables. Results show that PVT modules exhibit a small electrical efficiency reduction due to increased cell temperatures, which is largely compensated by the simultaneous thermal generation, yielding overall efficiency gains of 74.04%, 76.53%, and 7.62% over the reference PV system for Normal, Forced, and Closed circulation, respectively. The comparative analysis identifies Forced-circulation scheduling and the matching between thermal generation and consumption as key factors for performance optimization. The findings provide practical guidelines for deploying PVT systems to replace electric showers in tropical regions, reducing residential electricity consumption and mitigating peak-demand stress on the grid.

## 1. Introduction

In the pursuit of expanding access to clean and affordable energy, the increased deployment of distributed generation (DG) systems based on photovoltaic (PV) technology has emerged as a prominent solution because of its cost competitiveness, low emissions, and reduced maintenance requirements [1]. However, only a small fraction of incident solar irradiation is converted into electricity by PV cells, with the remainder dissipated as heat, which ultimately reduces module efficiency [2]. Hybrid photovoltaic–thermal (PVT) systems address this limitation by coupling a PV module to a flat-plate heat exchanger, enabling the simultaneous generation of electrical and thermal energy from the same collector area [3]. Since their introduction in 1973 [4], PVT systems have evolved into mature configurations operating with air or water as the working fluid, the latter being particularly suitable for tropical climates and residential applications such as domestic hot water supply [5,6]. Reported overall efficiencies range from 70% to 80% [7,8], and water-based PVT systems can reduce both the installation footprint and the payback period when compared to conventional PV systems [9], while also alleviating peak grid demand by displacing electric water-heating loads [10].

Despite this potential, the actual performance of PVT systems is strongly conditioned by the balance between thermal generation and consumption. When the produced heat is not effectively used, it accumulates in the storage tank, raising the working-fluid temperature and progressively reducing both heat-transfer effectiveness and PV electrical efficiency [11]. The choice between natural (thermosiphon) and forced (pumped) circulation also plays a critical role, since pumped configurations achieve higher heat transfer at the cost of additional electrical consumption, and should ideally operate only when there is an actual thermal demand [10]. These operational trade-offs become particularly relevant in tropical-climate countries, where stable irradiation and the absence of freezing conditions favor PVT deployment [12], yet adoption remains marginal. In Brazil, for instance, water heating accounts for approximately 13% of residential electricity consumption [13], reaching up to 24% in lower-income households [14] and 28% in specific regions [15], with electric showers being the dominant load and a major contributor to peak-demand stress on the grid. Nonetheless, the country reported less than 26 m^2^ of installed PVT area in 2023 [16,17], evidencing a clear mismatch between technical potential and effective deployment.

Research on PVT systems has advanced along three complementary fronts. The first concerns material and design innovations aimed at improving heat extraction: Phase-Change Materials (PCMs) and nanofluids [11], PCM-integrated absorbers achieving up to 74.1% efficiency [18] and 1.58-year payback [19], geometric optimizations such as serpentine and “S”-shaped channels [20,21,22], and advanced absorber concepts including petal arrays with nano-enhanced PCMs that increased total energy production by up to 27.09% [23] have all been proposed to enhance thermal transfer and reduce cell temperature. Comparative analyses of working fluids have also shown that water-based systems achieve higher thermodynamic performance than oil-based configurations [24]. The second front addresses regional and climatic adaptation, with studies confirming the effectiveness of water cooling in hot climates [25], the influence of module brand on tropical performance [26], and the integration of thermoelectric generators for additional gains [27]. A third line focuses on system-level operation, including affordable plastic-pipe configurations [28], dual oscillating absorbers [5,29], industrial drying applications [30], and frameworks for nighttime and transient behavior [31]. Recent reviews [32,33,34] consolidate these advances and project significant emission reductions through wider PVT adoption. Despite the growing body of knowledge, experimental studies involving PVT systems based on commercial photovoltaic modules coupled to roll-bond heat exchangers, including a storage tank and a consumption point (shower), remain scarce. This is particularly evident for investigations that compare multiple operating regimes of the same PVT installation under real outdoor conditions, as well as their direct comparison with a co-located conventional PV system. This gap is even more significant in tropical climates and in studies with comprehensive instrumentation of thermal, electrical, and meteorological variables.

To address this gap, this work presents an experimental evaluation of a modular water-based PVT system installed in São Mateus, Brazil, operated under three distinct hydraulic regimes—Normal (thermosiphon), Closed (no circulation), and Forced (pumped) circulation—and compared against a co-located conventional PV reference system. The novelty of the present study lies in the combination of (i) use of a modular PVT system coupled to commercial photovoltaic modules, with a storage tank and a consumption point, (ii) a fully instrumented modular PVT installation operating under real tropical conditions, (iii) a dedicated real-time data acquisition system based on an ESP32-C6 microcontroller monitoring thermal, electrical, and meteorological variables, and (iv) a direct multi-mode comparison that quantifies the electrical and thermal trade-off intrinsic to PVT operation. The specific objectives are therefore to (a) characterize the energetic performance of each operational mode under equivalent solar resource conditions, (b) quantify the electrical penalty and the thermal benefit of PVT operation relative to a conventional PV baseline, and (c) identify the operational strategy that maximizes the overall system efficiency for residential applications, with particular attention to the Brazilian scenario, where displacing electric showers by solar-heated water represents a tangible contribution to grid stability and household energy efficiency. The remainder of the paper is organized as follows: Section 2 describes the materials and methods; Section 3 presents the experimental results across the three operating modes; Section 4 discusses and compares the multi-day campaign results; and Section 5 summarizes the main conclusions and outlines directions for future work.

## 2. Materials and Methods

Photovoltaic systems generate electrical energy by converting solar radiation into electricity through modules composed of multiple photovoltaic cells. These cells, manufactured from semiconductor materials, operate based on the photoelectric effect, absorbing photon energy and releasing electrons [35]. The electrical power generated, $P_{el}$ (W), depends directly on the incident solar irradiance, *G* (W/m^2^), and the module area, *S* (m^2^). However, photovoltaic modules are highly sensitive to temperature, with performance decreasing as cell temperature increases [25]. A PVT module, in addition to electrical generation, absorbs part of the thermal energy from solar radiation through a heat exchanger attached to its backsheet. The useful thermal energy, $Q_{u}$ (W), can be used in low-temperature applications (<60 °C), reducing electrical energy consumption for water heating [2].

The present study employs a modular PVT system coupled to a commercial photovoltaic module, integrated with a storage tank and a consumption point, simulating a real application of the recovered thermal energy. Through an instrumentation system based on the ESP32 (Espressif Systems 32-bit microcontroller, Shanghai, China), electrical, thermal, and environmental variables are monitored and used for comparison with a conventional PV system, in which the PVT system is evaluated under three different water circulation modes, while both systems are maintained under the same environmental conditions.

### 2.1. PV and PVT Experimental Setup

Developed in the Renewable Energy Laboratory of the Federal Institute of Espírito Santo (IFES), São Mateus campus, this study employs an experimental plant composed of a photovoltaic (PV) system and a photovoltaic–thermal (PVT) system operating under the same environmental conditions.

**Figure 1:**
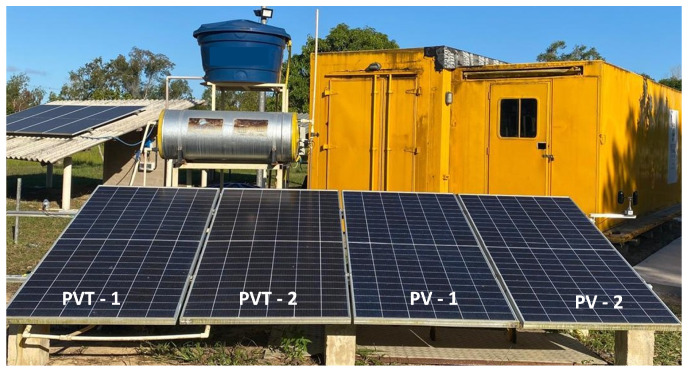
PV and PVT systems used in the experimental setup.

The PV and PVT plants consist of four identical photovoltaic modules connected to an APsystems QS1A microinverter, rated at 1500 W and equipped with four independent input channels, thereby simulating four distinct power generation units. Two modules operate as a conventional PV system, while the other two are adapted with heat exchangers attached to their backsheets, through which water circulates as the working fluid. The PV system is composed of two Canadian Solar modules (model CS3W-425), each with a nominal peak power of 425 W. Each module has a total area of 2.209 m^2^ and an effective area of 1.984 m^2^, and they are installed at a tilt angle of 19° with an azimuth angle of 0° (north-facing). Figure 1 shows the plant used in the study.

**Figure 2:**
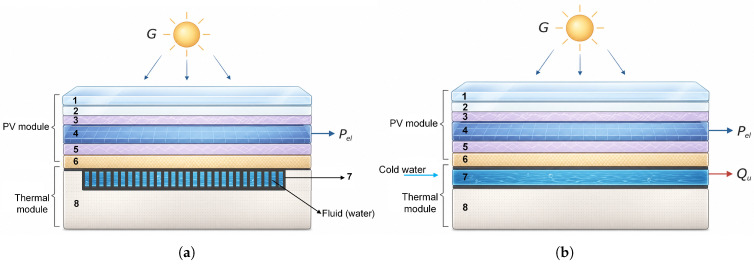
Layers of a PVT system with flat roll-bond tubes: (**a**) horizontal cross-section; (**b**) vertical cross-section. Layers: (1) glass; (2) air; (3) EVA (Ethylene Vinyl Acetate); (4) PV cells; (5) EVA; (6) Tedlar; (7) roll-bond tube heat exchanger; (8) insulating layer.

The PVT system employs the same modules, installed with the same tilt angle. Heat exchangers are attached to the backsheet of each module, providing a total heat exchange area of 1.8 m^2^. The flat-plate heat exchanger installed on both modules is of the roll-bond tube type, with the main advantage of retrofitting, enabling the conversion of conventional PV systems into PVT systems. Figure 2 presents the horizontal and vertical cross-sections of the photovoltaic–thermal (PVT) module adapted from a PV module, along with the description of each constituent layer.

The hydraulic circuit of the PVT system operates in a manner equivalent to a real installation, in which water from a reservoir at ambient temperature is heated in the heat exchangers and stored in a boiler. The hot water used in the shower simulation is replaced by water from the reservoir, ensuring that the hydraulic system operates with a constant total water volume. Comprising five valves that control and direct water flow, the system can simulate different operating conditions.

**Figure 3:**
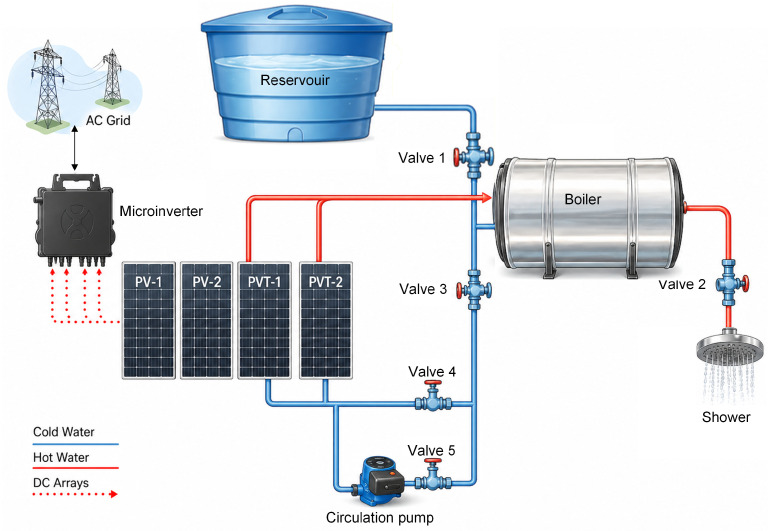
Schematic representation of the experimental PV and PVT systems.

Valves 1 and 3 provide hydraulic isolation of the boiler, allowing it to be decoupled from the main circuit for maintenance, system filling, or specific operating conditions. Valve 2 regulates the outlet of hot water from the boiler to the consumption point (shower), controlling the supply of heated water and enabling isolation of the consumption line when required. Valves 4 and 5 control the circulation regime of the working fluid within the thermal circuit, allowing operation under natural thermosiphon circulation or Forced circulation through a Komeco TP20-100W hydraulic circulation pump, respectively. In this way, the system can operate under different hydraulic and thermal conditions, enabling evaluation of the PVT system performance under distinct operating regimes. Figure 3 presents a detailed view of the PV and PVT systems that comprise the experimental plant.

### 2.2. Data Acquisition

To compare the performance of PV and PVT systems, data on electrical, thermal, and environmental conditions are required. For this purpose, an instrumentation system based on the ESP32 microcontroller was developed to measure these variables at one-minute intervals and transmit the data to an online database for further analysis. The system measures 16 variables, as listed in Table 1, including their respective symbols and units.

**Figure 4:**
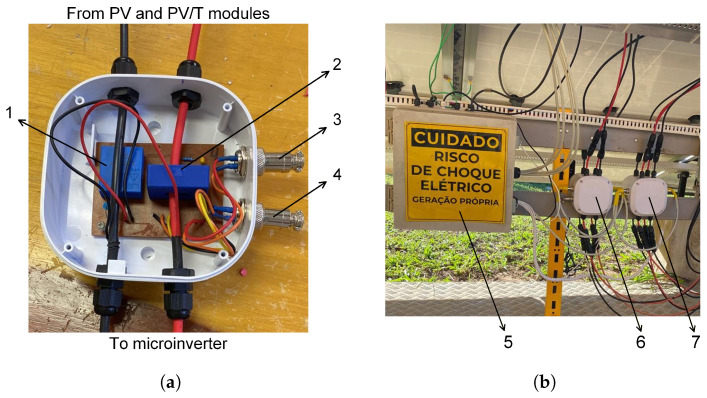
Voltage and current data acquisition sensors used in the experimental setup. (**a**) Internal view of the protection box containing the printed circuit board (PCB): (1) voltage sensor; (2) current sensor; (3) circuit power supply input; (4) sensor signal output. (**b**) Installation of the sensors beneath the photovoltaic modules: (5) enclosure containing the microcontroller and connection point for the remaining sensors; (6) sensors of the PV1 and PV2 modules; (7) sensors of the PVT1 and PVT2 modules.

For voltage measurement in each module, the LV 20-P sensor from LEM International was used. This sensor operates based on the Hall effect and features a measurement range from 10 to 500 V, an accuracy of ±1%, and linearity better than 0.2% [36]. The selected current sensor was the LA 55-P model, also manufactured by LEM International and based on the Hall effect, with an accuracy of ±0.65% [37] and linearity better than 0.15%. Both sensors provide current output signals and were mounted on a printed circuit board (Figure 4a), which was directly connected to the modules and the microcontroller (Figure 4b). The output currents from each sensor were converted into voltage signals using precision resistors, according to the manufacturers’ specifications, enabling proper signal conditioning and processing by the microcontroller for electrical power calculation.

Solar irradiance is measured using the SR05-D1A3 sensor, a pyranometer with a linearity of ±1% and a measurement range of 0 to 1600 W/m^2^. This sensor generates an output voltage ranging from 0 to 1 V, which is used to calculate electrical and thermal efficiencies.

Temperature measurements in the system are performed using DS18B20 digital thermometers from Dallas Semiconductor. These sensors employ the 1-Wire communication protocol, configured at 12-bit resolution, with an accuracy of ±0.125 °C [38]. In total, five temperature sensors are installed at different points in the system: ambient air, boiler, heat exchanger outlet, storage tank, and heat exchanger inlet. The data from each sensor is decoded and processed by the microcontroller using a dedicated software library.

The flow rate is measured using an ultrasonic sensor, model ECR MTDS-100F, which provides a current output signal ranging from 0 to 20 mA. This signal is converted into a voltage signal (0–3 V) using precision resistors. In addition, wind speed measurements, which can be used in system modeling, were performed using an AN-4A anemometer with a measurement range of 0 to 42 m/s and an output signal ranging from 0 to 10 V.

**Figure 5:**
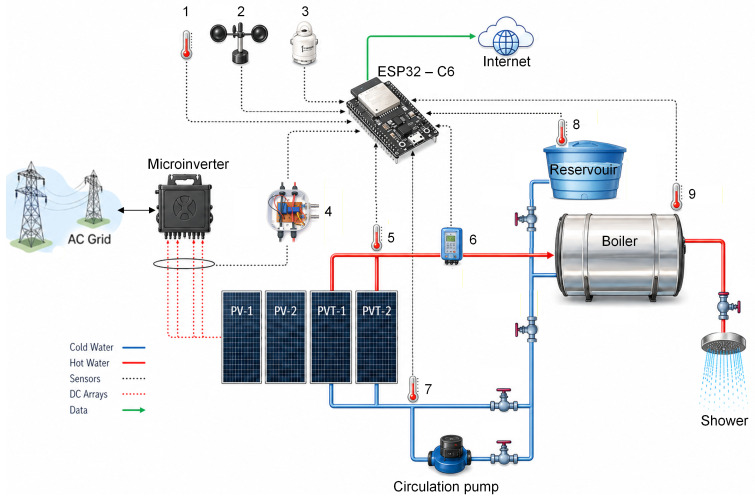
Data acquisition system and respective installed sensors: (1) ambient temperature ($T_{amb}$); (2) wind speed ($V_{wind}$); (3) solar irradiance (*G*); (4) voltage and current sensors (V/I); (5) PVT outlet temperature ($T_{o}$); (6) mass flow rate ($\dot{m}$); (7) PVT inlet temperature ($T_{i}$); (8) reservoir temperature ($T_{res}$); (9) boiler temperature ($T_{boiler}$).

The microcontroller used for processing the sensor signals is an ESP32-C6, responsible for reading all variables, calculating the electrical power based on the measured voltage and current values, and transmitting the data as an array. To improve the resolution of the measured analog quantities and mitigate inherent linearity errors of the ESP32-C6, an analog-to-digital converter (ADC), the ADS1115, was employed. This device features four 16-bit channels and high precision. The ESP 32-C6 was programmed to communicate with the Firebase platform, a Google-developed development environment that enables real-time data storage and monitoring. Although Firebase is primarily intended for application development rather than as a conventional database, it offers free access and user authentication and permission control features, which motivated its selection. Figure 5 shows the schematic interconnection between the sensors, the microcontroller, and the PV and PVT systems.

The estimated uncertainty values for the sensors used in the data acquisition system, considering both the manufacturer-declared calibration accuracy and the resolution of the 16-bit analog-to-digital converter (ADC) of the ESP32-C6 microcontroller, are presented in Table 2. For each sensor, the calibration uncertainty ($u_{cal}$), the quantization uncertainty ($u_{q}$), the combined standard uncertainty ($u_{c}$) and the expanded uncertainty (*U*, k=2) are reported in the corresponding measurement units, together with the relative expanded uncertainty (U%).

In order to mitigate short-term fluctuations in the acquired signals, the data acquisition system samples each sensor twenty times and computes a moving average, providing a more representative reading for every monitored quantity. The sensors were installed following the manufacturer’s guidelines, and shielded cables were adopted throughout the wiring to attenuate electromagnetic interference.

### 2.3. Experimental Procedure

To evaluate the energy gain of the PVT system relative to the conventional PV system, data were collected under three operating regimes: Normal water circulation, Forced water circulation, and closed-loop circulation. These operating modes were selected to assess the influence of different hydraulic configurations on the thermal and electrical performance of the PVT system. Each operational regime was simulated by combining valve openings and closures within the hydraulic circuit. Table 3 presents the operating conditions of each valve for the selected regimes.

The Normal circulation regime simulates the operation of a residential system in which hot water is consumed and replenished from a reservoir, while water circulation through the heat exchanger occurs naturally via the thermosiphon effect. In this configuration, the heated water is stored in a boiler for subsequent use, such as in showers. During this test, Valve 02 was opened at 7:00 a.m. for 20 min, simulating domestic hot water consumption during a shower.

Under the Closed circulation regime, the heated water is not consumed throughout the day and continuously circulates exclusively between the heat exchanger and the boiler, simulating a residential scenario with little or no hot water consumption.

For the Forced circulation regime, the same operating conditions as the Normal circulation regime are maintained; however, water circulation is driven by a hydraulic pump. Similarly to the Normal circulation cycle, a hot water consumption event was simulated for 20 min, starting at 7:30 a.m. At 9:00 a.m., the hydraulic circulation pump was activated, imposing a flow rate of approximately 7.65 L/min through the heat exchanger circuit, and was deactivated at 4:00 p.m.

For each operating regime, electrical output power, temperatures, solar irradiance, and water flow rate were continuously monitored from 4:00 a.m. to 6:30 p.m., with one-minute sampling intervals. To evaluate the temporal and quantitative performance of each system and to generate performance and comparative graphs, MATLAB R2025a (MathWorks Inc., Natick, MA, USA) and the Matplotlib 3.10.8. library for Python 3.11 were used.

A temporal analysis was conducted using single-day data to characterize the thermal and electrical behavior of the PVT system and evaluate its daily performance. The analysis was based on the heat exchanger inlet ($T_{i}$) and outlet ($T_{o}$) temperatures, the ambient temperature ($T_{amb}$), the boiler temperature ($T_{boiler}$), and the electrical power output of each of the four modules. Additionally, instantaneous and average electrical efficiency ($\eta_{el}$, %), overall efficiency ($\eta_{ov}$, %), and total energy yield were evaluated over the monitoring period.

The performance of the three circulation modes was compared through a quantitative analysis based on the mean electrical efficiencies, $\bar{\eta }el{,}_{PV}$ (%) and $\bar{\eta }el{,}_{PVT}$ (%), the mean thermal efficiency, $\bar{\eta }th$ (%), and the mean overall efficiency, $\bar{\eta }ov$ (%), as well as the environmental parameters, including daily irradiation (*H*, kWh/m^2^), and mean irradiance (*G*, W/m^2^). All metrics were calculated from datasets comprising six days for the Forced and Closed modes, and five days for the Normal mode. The analysis was carried out using established statistical methods, including arithmetic mean as a measure of central tendency, sample standard deviation to assess data dispersion, and the estimation of 95% confidence intervals based on the Student’s t-distribution, allowing the quantification of experimental variability and ensuring the robustness of the results.

### 2.4. Performance Metrics and Data Analysis

The electrical efficiency of a photovoltaic module, $\eta_{el}$ (%), can be calculated from instantaneous values of electrical power, $P_{el}$ (Watts, W), and solar irradiance, *G* (W/m^2^):

(1) $$\eta_{el}\left(t\right)=\frac{P_{el}\left(t\right)}{G(t)\cdot S}=\frac{V(t)\cdot I(t)}{G(t)\cdot S}$$

where *V* (Volt, V) and *I* (Ampere, A) are the voltage and current generated by the module, respectively, and *S* (m^2^) is its area. The integrated mean electrical efficiency, ${\bar{\eta }}_{el}$ (%), is defined as the ratio between the time-integrated electrical power output and the time-integrated incident solar energy.

According to [25], the main factors that directly affect the thermal performance of PVT systems are solar irradiance, ambient temperature, fluid inlet and outlet temperatures, wind speed, and liquid flow rate. The combination of these factors, together with the thermal resistances of the system layers, results in a complex mathematical model, as described in [8]. To calculate the thermal energy collected by the heat exchanger, $Q_{u}$ (W), a simplified approach described by [39] was adopted, relating the mass flow rate, $\dot{m}$ (kg/s), the specific heat capacity of water, $C_{p}$ (4186 J/(kg·K)), and the system thermal gradient, defined as the difference between the inlet temperature, $T_{i}$ (°C), and outlet temperature, $T_{o}$ (°C), as expressed in Equation (2). The mass flow rate is computed from the volumetric flow rate (L/s), assuming a water density of 1000 kg/m^3^.

(2) $$Q_{u}=\dot{m}\cdot C_{p}\cdot \left(T_{o}-T_{i}\right)$$

The instantaneous thermal efficiency of the system, $\eta_{th}$ (%), is defined as the ratio between the instantaneous thermal energy collected by the heat exchanger, $Q_{u}$ (W), and the product of the instantaneous solar irradiance, *G* (W/m^2^), and the collector area, *S* (m^2^) [40].

(3) $$\eta_{th}=\frac{Q_{u}\left(t\right)}{G(t)\cdot S}$$

Similarly to the integrated mean electrical efficiency, the integrated mean thermal efficiency, ${\bar{\eta }}_{th}$ (%), is defined as the ratio between the time-integrated useful thermal energy and the time-integrated incident solar energy.

From a thermodynamic perspective, thermal and electrical energy are distinct forms of energy. However, a widely adopted simplification for calculating the instantaneous overall efficiency of a PVT system, $\eta_{ov}\left(t\right)$ (%), can be expressed by Equation (4) [4]. The integrated mean overall efficiency of the PVT system, ${\bar{\eta }}_{ov}$ (%), was obtained by summing the integrated mean electrical and thermal efficiencies.

(4) $$\eta_{ov}\left(t\right)=\eta_{el}\left(t\right)+\eta_{th}\left(t\right)$$

## 3. Results

### 3.1. Temporal Analysis

The temporal analysis presents the variation of temperatures, generated power, and efficiency throughout a single day, based on instantaneous measurements recorded at 60 s intervals. The temporal evolution of the ambient temperature ($T_{amb}$, °C), the heat exchanger inlet ($T_{i}$, °C) and outlet ($T_{o}$, °C) temperatures, and the boiler temperature ($T_{boiler}$, °C) can be observed, as well as the electrical power generated by each PV module ($P_{PV1}$, $P_{PV2}$, W) and PVT module ($P_{PVT1}$, $P_{PVT2}$, W). In addition, the evolution of the instantaneous overall efficiency ($\eta_{ov}\left(t\right)$, %) of the PVT system and electrical efficiency ($\eta_{el}\left(t\right)$, %) of PV system can be followed throughout the day. These results are presented as plots of temperature, electrical power, and efficiency, enabling performance analysis of the three operating modes evaluated in this study.

#### 3.1.1. Normal Circulation

Under Normal circulation, the temperature of the water stored in the boiler, initially 35.25 °C, decreased to 27.97 °C after hot water consumption, reaching a value close to the ambient temperature. As solar irradiance and ambient temperature increase throughout the day, the temperature gradient also increases, which is defined as the difference between the inlet and outlet temperatures of the heat exchanger. Consequently, the PVT thermal generation also increases to heat the water accumulated in the boiler.

**Figure 6:**
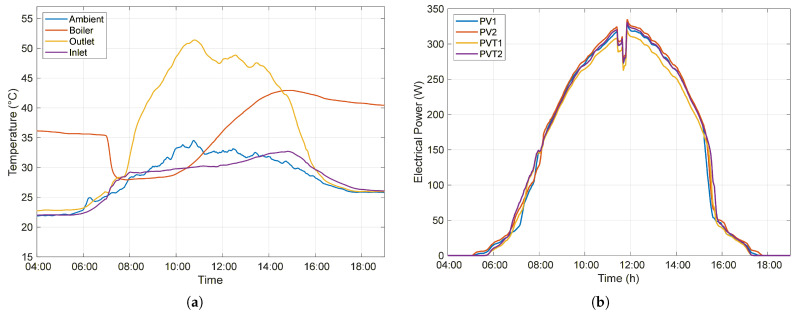
Performance of the PV and PVT systems under Normal circulation conditions: (**a**) temporal evolution of temperatures (ambient, boiler, inlet, and outlet of the heat exchanger); (**b**) electrical power generated by the PV ($P_{PV1}$, $P_{PV2}$) and PVT ($P_{PVT1}$, $P_{PVT2}$) modules throughout the day.

At 2:50 p.m., the water temperature in the boiler reached 42.94 °C, at which point the system reached thermal equilibrium. From this moment onward, the outlet water temperature of the heat exchanger became lower than the temperature of the water stored in the boiler, causing the system to lose thermal energy, since the water continued to circulate through the system while returning to the storage tank at a temperature lower than that of the stored fluid. The rate of temperature decay of the stored water decreased after 4:00 p.m., when the temperature gradient decreased, leading to reduced heat loss to the environment. Figure 6a illustrates the behavior of the monitored temperatures throughout the day for the Normal operating cycle.

The PV system generated 4.234 kWh of electrical energy over the day, with an average efficiency of 15.39%. On the other hand, the PVT system accumulated 4.185 kWh (15.21% on average), a small reduction of 1.16%. For most of the day, between 9:00 a.m. and 3:00 p.m., the PV modules produced slightly higher power than the PVT modules. Figure 6b shows the instantaneous electrical power generated by each of the four modules throughout the day.

Despite exhibiting lower electrical efficiency throughout the day, the PVT system achieves higher overall efficiency due to the simultaneous contribution of electrical and thermal components, as defined by the total efficiency (Equation (4)). While the PV system converts only a fraction of the incident irradiance into electrical energy, the PVT system additionally exploits the thermal energy extracted from the photovoltaic module by the working fluid, 10.19% on average, significantly enhancing the overall energy efficiency to 25.40%.

**Figure 7:**
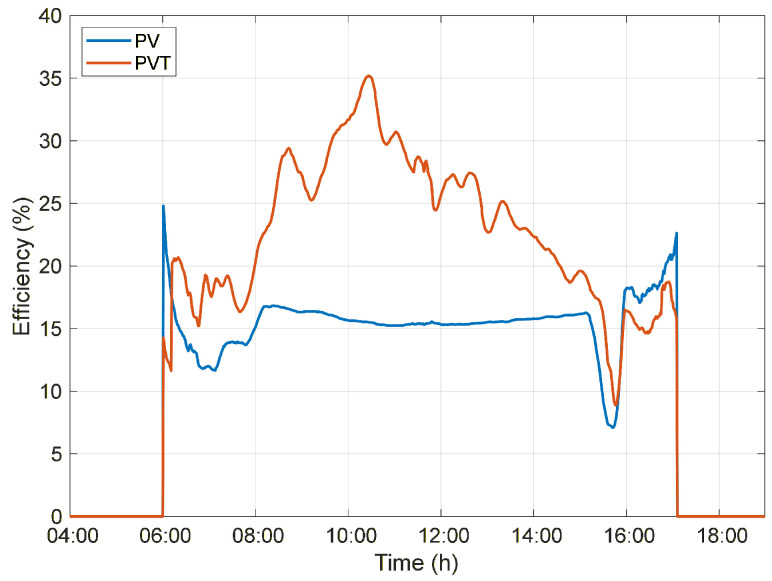
Overall efficiency of the PV and PVT systems throughout the day under Normal circulation conditions.

During the morning period, when the system’s water temperature remains relatively low, the temperature gradient is high, leading to increased thermal energy absorption. This behavior explains the peaks observed in the instantaneous overall efficiency of the PVT system, which reaches a maximum value of 35.18% at 10:26 a.m., as shown in Figure 7. In contrast, the PV system maintains a nearly constant instantaneous efficiency of 15–17%, as it is limited exclusively to electrical energy conversion.

#### 3.1.2. Closed Circulation

Under Closed-circulation conditions, with no consumption of heated water throughout the day, the water continuously circulates within the system. During the early hours of the day, the initial water temperature in the boiler (37.69 °C) is higher than the ambient temperature (30 °C), causing the PVT system to lose heat to the environment, which in turn reduces the boiler temperature.

**Figure 8:**
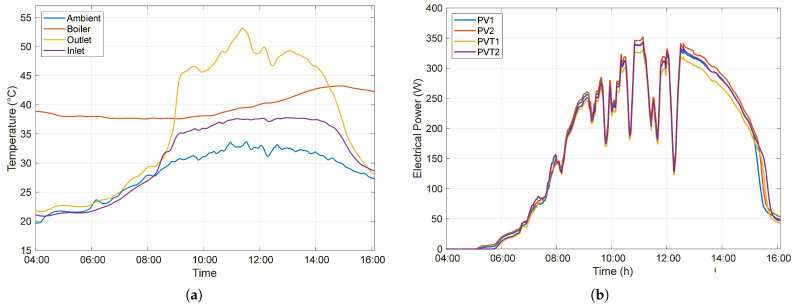
Performance of the PV and PVT systems under Closed circulation conditions: (**a**) temporal evolution of temperatures (ambient, boiler, inlet, and outlet of the heat exchanger); (**b**) electrical power generated by the PV ($P_{PV1}$, $P_{PV2}$) and PVT ($P_{PVT1}$, $P_{PVT2}$) modules throughout the day.

As the temperature irradiation increases, the heat exchanger absorbs thermal energy, and the water temperature in the boiler gradually rises, reaching a maximum value of 43.25 °C at 2:49 p.m. From this point onward, as the outlet temperature of the heat exchanger decreases, the water returning to the boiler becomes cooler than the previously stored water, resulting in thermal energy losses within the system. After 4:00 p.m., as the temperature gradient approaches zero, the rate of decrease in the stored water temperature decreases. The daily profiles of the ambient temperature, the inlet and outlet temperatures of the heat exchanger, and the boiler water temperature under Closed-circulation conditions are presented in Figure 8a.

The lack of utilization of the thermal energy accumulated by the system results in the water temperature at the heat exchanger inlet being higher than the ambient temperature. Consequently, the photovoltaic cells of the PVT modules operate at higher temperatures than those of the PV system, directly affecting their electrical efficiency. Due to the elevated cell operating temperature, the PVT system, which generated 4.053 kWh, exhibits an average production that is 2.65% lower than that of the PV system, which produced a total of 4.164 kWh. Figure 8b presents the instantaneous electrical power generated by each of the four modules over the day.

**Figure 9:**
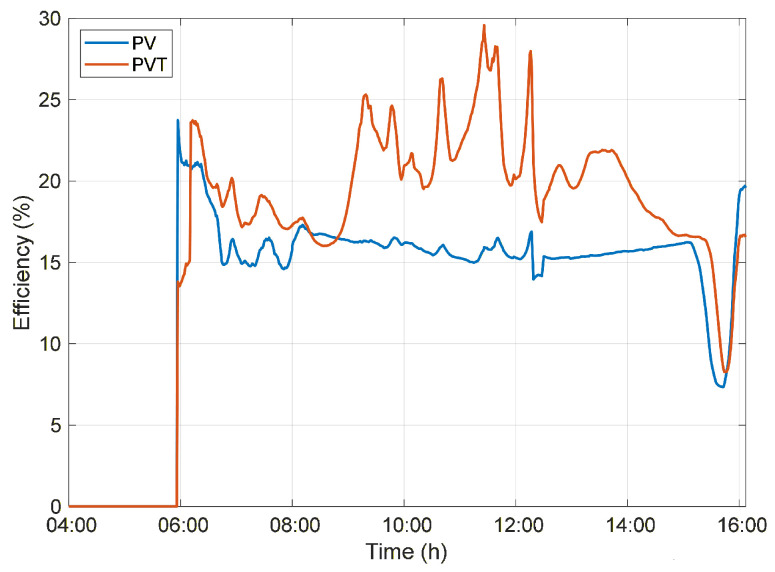
Overall efficiency of the PV and PVT systems throughout the day under Closed circulation conditions.

Throughout the day, the PVT system also exhibited higher overall efficiency, reaching maximum values above 25% between 9:00 a.m. and 12:30 p.m., while the PV system efficiency remained consistent with previous results, varying between 15% and 17%, as shown in Figure 9.

#### 3.1.3. Forced Circulation

Under Forced circulation, the temperature of the water stored in the boiler, initially 35.64 °C, decreased to 27.8 °C after the hot water was used, reaching a value close to the ambient temperature. With continuous circulation, the outlet temperature of the heat exchanger decreased rapidly until 9:10 a.m., while the temperature of water accumulated in the boiler increased rapidly. From this point onward, the temperature gradient remained nearly constant, allowing the boiler water temperature to reach a maximum value of 45.09 °C at 2:31 p.m.

**Figure 10:**
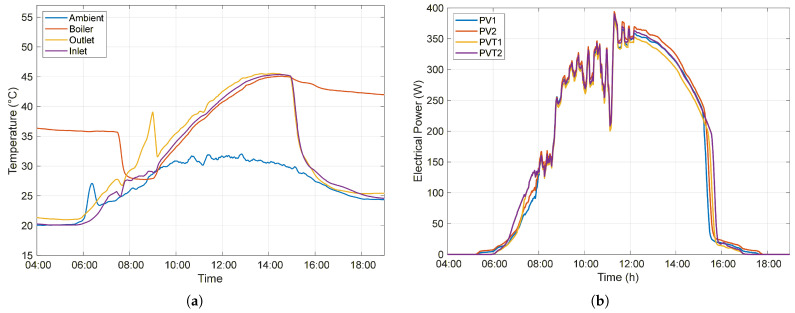
Performance of the PV and PVT systems under Forced circulation conditions: (**a**) temporal evolution of temperatures (ambient, boiler, inlet, and outlet of the heat exchanger); (**b**) electrical power generated by the PV ($P_{PV1}$, $P_{PV2}$) and PVT ($P_{PVT1}$, $P_{PVT2}$) modules throughout the day.

As observed in the other circulation modes, after reaching the maximum temperature at 2:31 p.m., the system began to lose thermal energy to the environment. Between 2:31 p.m. and 4:00 p.m., the high flow rate increased thermal losses, as reflected in the rapid decrease in boiler temperature. After 4:00 p.m., the pump was switched off, reducing the mass flow rate and, consequently, slowing the decline in boiler temperature due to reduced heat losses. Figure 10a illustrates the behavior of the monitored temperatures throughout the day for the Forced operating cycle.

The PVT system generated a total of 4.624 kWh, while the PV system produced 4.601 kWh, resulting in a 0.53% higher production than the PV system. The PVT electric generation is very close to that of the PV system between 8:00 a.m. and 12:00 p.m. Figure 10b presents the instantaneous electrical power generated by each of the four modules throughout the day.

**Figure 11:**
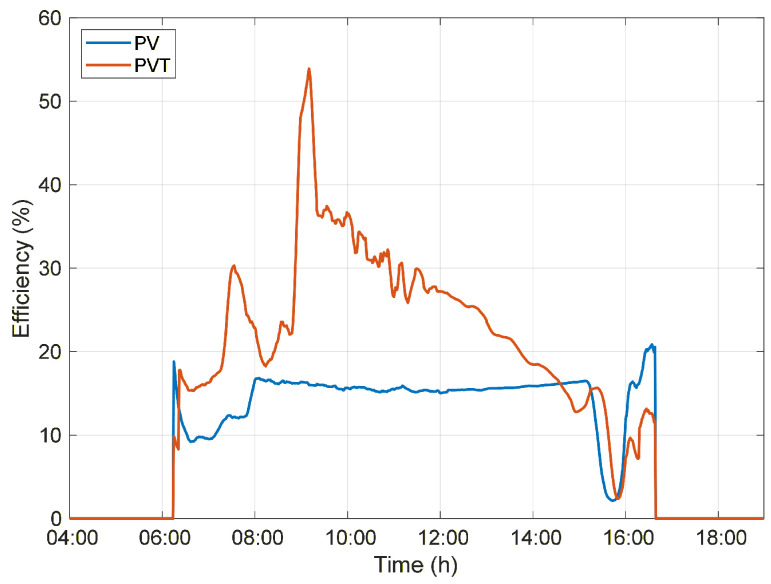
Overall efficiency of the PV and PVT systems throughout the day under Forced circulation conditions.

The increase in flow rate enhances heat transfer in the heat exchanger, increasing the rate of thermal energy removal from the photovoltaic module. As a result, a significant increase in thermal contribution to overall efficiency is observed, leading to an efficiency peak of approximately 53.9% at 9:10 a.m., as shown in Figure 11. After this peak, the overall efficiency of the PVT system gradually decreases, associated with a reduction in the temperature gradient and the system’s approach to thermal equilibrium, even under high flow rate conditions.

Nevertheless, for most of the operating period, the PVT system maintains a higher efficiency than the PV system. After the boiler water reaches its maximum temperature at 2:31 p.m., the outlet water temperature becomes slightly lower than the inlet temperature, and under high flow rate conditions, the system efficiency decreases as thermal energy losses to the environment increase, until the circulation pump is switched off.

In all three cases, instantaneous variations in the generated electrical power occur due to transient cloud cover (passing clouds), a common phenomenon in the tropical climate of São Mateus, Espírito Santo state, Brazil.

### 3.2. Quantitative Performance Analysis

**Figure 12:**
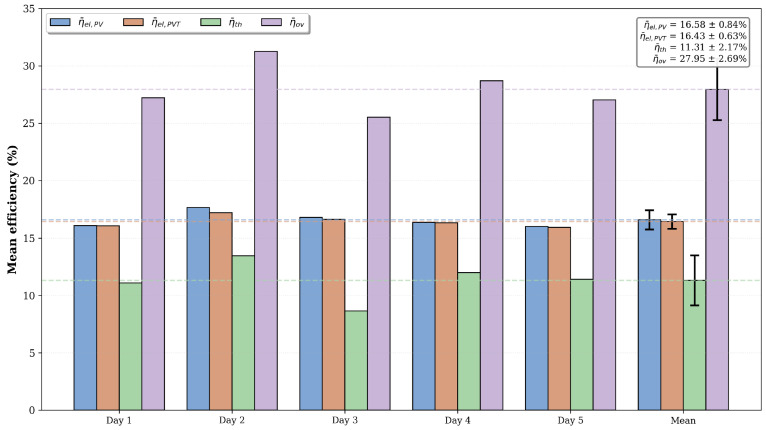
Mean electrical efficiency of the PV ($\bar{\eta }el{,}_{PV}$) and PVT ($\bar{\eta }el{,}_{PVT}$) systems, mean thermal efficiency ($\bar{\eta }th$), and mean overall efficiency ($\bar{\eta }ov$) over five days under Normal circulation conditions (dashed lines indicate the average values for each parameter).

Daily mean efficiencies were calculated through time-weighted averaging of instantaneous measurements (sampled at ∼60 s intervals), using the actual time step between consecutive data points to ensure accurate temporal integration. Campaign mean values represent the arithmetic mean across all test days, *n* (*n* = 5 for Normal, *n* = 6 for Closed and Forced), with 95% confidence intervals computed using Student’s t-distribution. Overall efficiency was defined as the sum of electrical and thermal efficiencies. Figure 12 shows the day-by-day performance variability for Normal mode, demonstrating consistent electrical efficiency (${\bar{\eta }}_{el}=16.43\pm 0.63\%$), thermal efficiency (${\bar{\eta }}_{th}=11.31\pm 2.17\%$), and overall efficiency (${\bar{\eta }}_{ov}=27.95\pm 2.69\%$) across the five-day measurement campaign.

Table 4 summarizes the campaign-averaged performance metrics for the three hydraulic operating modes, with all values reported as mean ± 95% confidence interval. Energy production metrics include the daily mean electrical energy from the PV reference ($E_{PV}$, in kWh), electrical energy from the PVT system ($E_{PVT}$, in kWh), thermal energy extracted by the collector ($Q_{th}$, in kWh), and equivalent total energy ($E_{equiv}$, in kWh) computed as the sum of electrical and thermal outputs. Mean efficiencies are reported for electrical conversion in both configurations (${\bar{\eta }}_{el,PV}$ and ${\bar{\eta }}_{el,PVT}$, in %), thermal conversion (${\bar{\eta }}_{th}$, in %), and overall system performance (${\bar{\eta }}_{ov}$, in %). Performance gains quantify the relative change of PVT compared to PV-only operation: $\Delta_{el}$ (in %) represents the electrical gain/loss defined as $\left(E_{PVT}-E_{PV}\right)/E_{PV}\times 100\%$, while $\Delta_{tot}$ (in %) represents the total energy gain defined as $\left(E_{equiv}-E_{PV}\right)/E_{PV}\times 100\%$. Environmental conditions and quality metrics include daily integrated irradiance (*H*, in kWh/m^2^), and mean in-plane irradiance ($\bar{G}$, in W/m^2^).

The table clearly shows that Forced mode achieves superior performance across all categories, with the highest thermal efficiency (13.67 ± 2.10%), overall efficiency (31.81 ± 4.37%), total energy gain (+76.53 ± 14.73%), and the only positive electrical gain (+0.70 ± 1.50%). In contrast, the Closed mode exhibits the poorest performance with minimal thermal recovery (4.89 ± 4.02%) and substantial electrical penalty (−6.69 ± 3.22%), while the Normal mode demonstrates balanced performance with good thermal efficiency (11.31 ± 2.17%) at the cost of a small electrical loss (−0.64 ± 1.20%).

## 4. Discussion

The multi-day experimental campaigns enabled a robust statistical comparison of the three hydraulic operating modes by averaging out day-to-day fluctuations in solar irradiance and ambient temperature. The campaign periods were conducted under comparable environmental conditions, with daily integrated irradiation ranging from 5.151 ± 0.468 kWh/m^2^ (Closed) to 6.240 ± 0.726 kWh/m^2^ (Normal) and mean irradiance between 554 ± 50 W/m^2^ (Closed) and 621 ± 78 W/m^2^ (Forced), as reported in Table 4.

### 4.1. Energy Production Across Operating Modes

**Figure 13:**
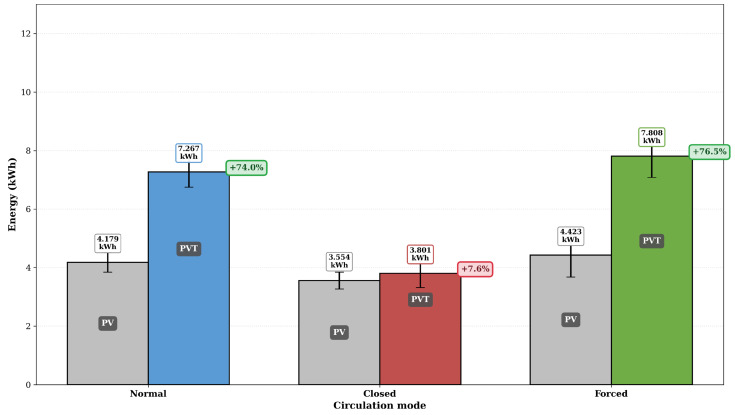
Comparison of the total equivalent energy ($E_{equiv}$) delivered by the PVT system relative to the PV reference under the three hydraulic operating modes. The percentage values represent the total energy gain of the PVT system over the PV reference.

When both electrical and thermal contributions are accounted for, the advantage of the PVT system over the conventional PV system becomes substantial. Figure 13 shows that the PVT system delivered total energy gains of +74%, +7.6%, and +76.5% over the PV reference in the Normal, Closed, and Forced circulation modes, respectively.

**Figure 14:**
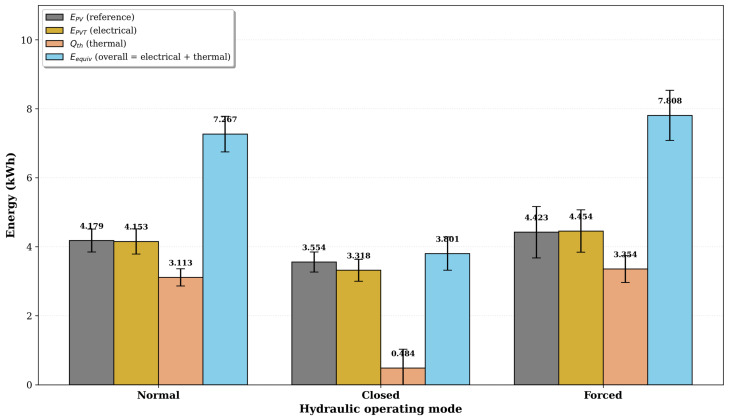
Daily mean energy production for each operating mode: electrical energy from PV reference ($E_{PV}$), electrical energy from PVT system ($E_{PVT}$), thermal energy recovered ($Q_{th}$), and equivalent total energy ($E_{equiv}$). Error bars represent 95% confidence intervals.

Figure 14 presents the daily energy production for each operating mode, including electrical energy from the PV reference ($E_{PV}$), electrical energy from the PVT system ($E_{PVT}$), thermal energy recovered ($Q_{th}$), and the equivalent total energy ($E_{equiv}$). The thermal energy production varied significantly across the three regimes, ranging from 0.484 ± 0.546 kWh in the Closed mode to 3.354 ± 0.389 kWh in the Forced mode, a nearly sevenfold difference. This variation highlights the hydraulic regime’s dominant role in determining the PVT system’s thermal performance.

In the Closed mode, the absence of hot water consumption causes the boiler to gradually reach thermal equilibrium, reducing the temperature gradient ($T_{o}-T_{i}$) and, consequently, the rate of useful heat extraction. In contrast, the Normal and Forced modes benefit from the periodic replenishment of cold water at the heat exchanger inlet, maintaining a higher temperature gradient throughout the day and enabling thermal energy outputs of 3.113 ± 0.250 kWh and 3.354 ± 0.389 kWh, respectively. The equivalent total energy reaches 7.808 ± 0.728 kWh in the Forced mode, slightly higher than the 7.267 ± 0.518 kWh delivered by the Normal mode, while the Closed mode yields only 3.801 ± 0.484 kWh.

**Figure 15:**
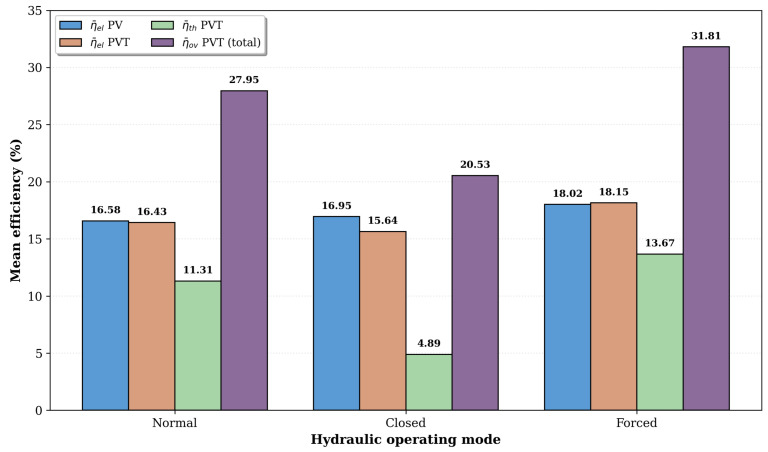
Mean electrical, thermal and overall efficiencies of the PVT system under the three hydraulic operating modes, compared with the reference PV system. The breakdown highlights the contribution of the thermal component to the overall efficiency gain.

The Forced circulation mode also achieved the highest mean overall efficiency (31.81%), followed closely by the Normal regime (27.95%) and, at a greater distance, by the Closed regime (20.53%), as shown in Figure 15.

Comparing the electrical energy generated under each circulation regime, it is observed that the heat absorbed by the working fluid increases the cell operating temperature, slightly reducing the electrical efficiency relative to the reference PV system. This effect was observed in the Normal and Closed circulation modes, with electrical losses of −0.64% and −6.69%, respectively. The greater reduction in the Closed regime is due to the fact that the heated water is not consumed, leading to a progressive accumulation of heat in the boiler and a higher inlet temperature at the heat exchanger.

**Figure 16:**
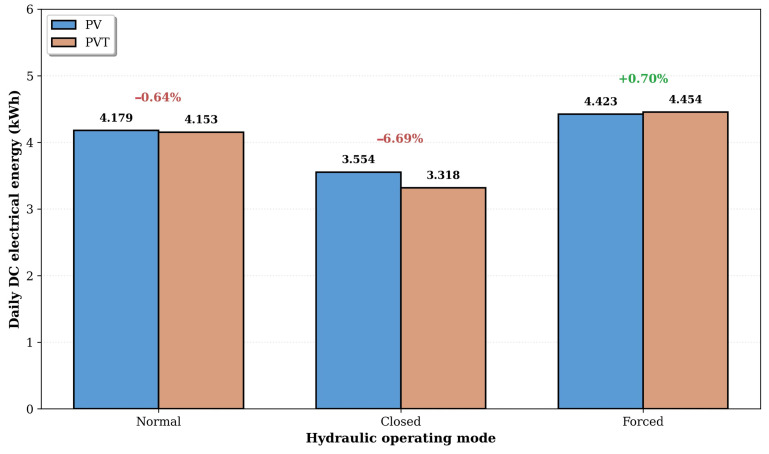
Comparison of the daily DC electrical energy generated by the PV and PVT systems under the three hydraulic operating modes. The percentage values represent the electrical gain (positive) or loss (negative) of the PVT system relative to the PV reference.

In the Forced circulation mode, however, the higher mass flow rate maintains a low inlet temperature throughout the day, enhancing heat removal from the back of the modules. Under these conditions, the PVT modules slightly outperformed the PV reference system (+0.70% in DC electrical energy), as shown in Figure 16, demonstrating that an actively cooled PVT module can simultaneously generate thermal energy and operate with electrical efficiency comparable to that of a conventional PV module.

An important observation from these results is that the difference between the Normal and Forced regimes in terms of overall efficiency is small (about 2.5 p.p.), while the Closed mode is significantly inferior to both. This confirms that, in the absence of an effective hot water demand, the additional cost of Forced circulation (due to pump electricity consumption) is not justified by a meaningful increase in efficiency, and a simple thermosiphon configuration can already provide satisfactory performance.

Forced circulation is particularly advantageous in installations with high thermal demand, such as residences with multiple bathrooms, hotels, and small commercial establishments, where the higher mass flow rate sustains the temperature gradient even under continuous hot water consumption.

On the other hand, in installations with low hot water consumption, the use of modular PVT systems is not recommended, as the limited thermal energy extraction can compromise overall performance, negatively affect electrical generation, and potentially reduce module lifetime due to increased photovoltaic cell temperatures.

### 4.2. Comparative Performance Assessment: Strengths and Weaknesses of Each Operating Mode

A comprehensive evaluation of the three hydraulic operating modes requires examining performance not only in individual metrics but also across the multidimensional space defined by electrical efficiency, thermal efficiency, overall efficiency, and energy gains. Each regime exhibits a unique combination of strengths and weaknesses that determine its suitability for different application contexts.

**Figure 17:**
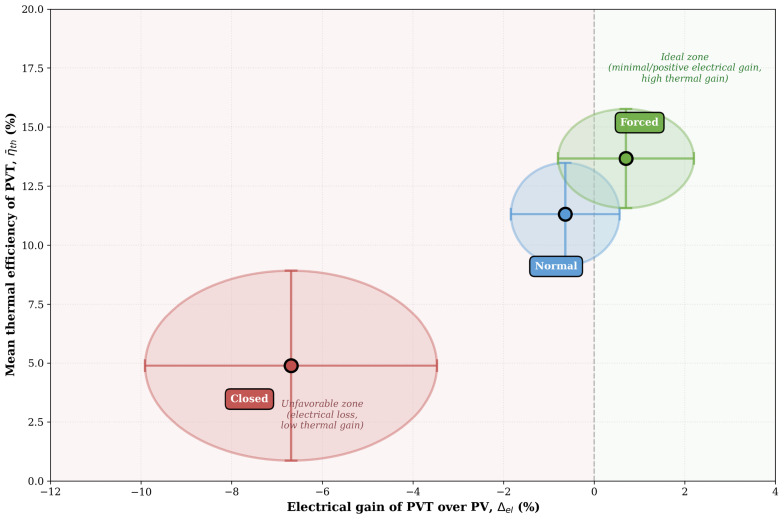
Electrical–thermal trade-off map for the three hydraulic operating modes tested over multi-day campaigns. Each point represents the mean performance with 95% confidence intervals shown as error bars and ellipses. The vertical dashed line at ($\Delta_{el}$) = 0% separates modes with electrical loss (left, red zone) from those with electrical gain (right, green zone).

Figure 17 presents the electrical-thermal trade-off map comparing the three hydraulic operating modes. The horizontal axis shows the electrical gain of the PVT system relative to the standalone photovoltaic reference ($\Delta_{el}$), while the vertical axis represents the mean thermal efficiency of the PVT collector (${\bar{\eta }}_{th}$). Each data point corresponds to the average performance over 5 to 6 days of testing, with 95% confidence intervals represented by error bars and ellipses.

The background shading delineates two performance zones: the unfavorable zone (red, left side, $\Delta_{el}<0\%$), where the PVT system exhibits electrical losses compared to PV-only operation, and the ideal zone (green, right side, $\Delta_{el}\geq 0\%$), where the PVT system maintains or improves electrical generation while simultaneously harvesting thermal energy.

The Forced circulation mode is the only configuration to reach the ideal zone, with a small but positive electrical gain (+0.70 ± 1.5%) and the highest thermal efficiency (13.67 ± 2.10%). The Normal mode operates close to the electrical break-even point, with minimal loss (−0.64 ± 1.20%) and good thermal recovery (11.31 ± 2.17%), representing a balanced trade-off.

In contrast, the Closed mode lies deep within the unfavorable zone, with significant electrical degradation (−6.69 ± 3.22%) and the lowest thermal efficiency (4.89 ± 4.02%). The high uncertainty in its thermal performance reflects inconsistent and inefficient heat extraction, resulting from the underutilization of the accumulated thermal energy (stored hot water) in the system.

**Figure 18:**
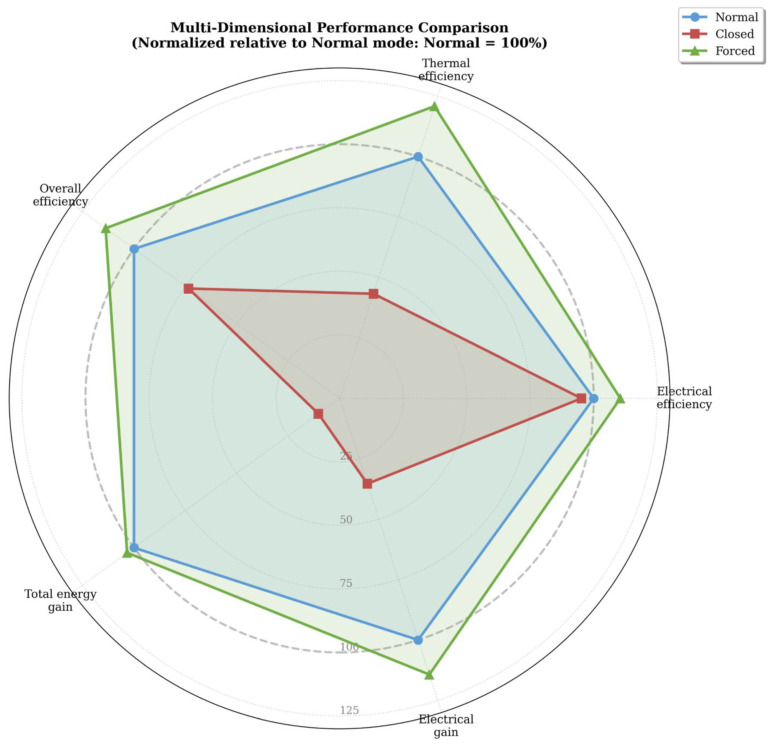
Multi-dimensional performance comparison showing each metric as percentage of Normal mode (100% baseline, dashed circle). Forced circulation achieves 10–21% gains across all metrics, while Closed circulation shows 57–90% degradation.

The multi-dimensional radar chart (Figure 18) provides a comprehensive visual comparison of the three hydraulic operating modes across five key performance metrics: electrical efficiency, thermal efficiency, overall efficiency, total energy gain, and electrical gain. To enable direct visual comparison, despite the different scales of these metrics (efficiencies ranging from 5–32% versus energy gains ranging from −7% to +77%), all values were normalized relative to the Normal mode, which serves as the 100% baseline reference. In this representation, values above 100% indicate superior performance compared to the Normal mode, while values below 100% indicate inferior performance. The resulting pentagonal shapes for each mode clearly illustrate their relative strengths and weaknesses across all performance dimensions simultaneously.

The Forced circulation mode (green) forms the largest pentagon, extending beyond the Normal mode baseline (dashed line at 100%) in all five dimensions, demonstrating superior performance across every evaluated metric. Specifically, the Forced mode achieves 110% of the Normal mode electrical efficiency (18.15% vs. 16.43%), 121% of thermal efficiency (13.67% vs. 11.31%), 114% of overall efficiency (31.81% vs. 27.95%), and 103% of total energy gain (76.53% vs. 74.04%), and reaches the ideal zone with positive electrical gain, whereas the Normal mode exhibits a small loss. This consistent outperformance confirms that Forced circulation with optimized flow rate not only maximizes thermal energy extraction but also enhances electrical generation through more effective cell cooling, effectively eliminating the traditional electro-thermal trade-off. The expansion of the pentagon in all directions confirms that Forced circulation represents a true Pareto improvement over natural circulation.

In contrast, the Closed circulation mode (red) forms a severely contracted pentagon that approaches the center across all dimensions, indicating systematic operational failure. This mode achieves only 95% of the Normal mode electrical efficiency, just 43% of thermal efficiency (4.89% vs. 11.31%, the most significant loss), 73% of overall efficiency, and only 10% of total energy gain. The electrical gain dimension also collapses completely, with the Closed mode exhibiting the largest electrical penalty (−6.69% relative to the Normal mode) among all configurations. This multi-dimensional collapse demonstrates that low fluid circulation (due to the lack of heated water) not only impairs thermal energy extraction but also degrades electrical performance by causing excessive cell temperatures. The visual compression of the red pentagon provides clear evidence that both forms of energy require active fluid circulation to achieve acceptable performance, and that stagnation leads to cumulative losses rather than trade-offs.

### 4.3. Summary of Findings

The comparative analysis presented in this section can be summarized in the following key points:

- Trade-off elimination demonstration: This work provides experimental evidence that the conventional electro-thermal trade-off in PVT systems can be eliminated through optimized Forced circulation, achieving simultaneous positive electrical gain (+0.70%) and maximum thermal efficiency (13.67%), challenging literature assumptions of inevitable electrical penalties.
- Identification of the compound loss mechanism: The results of the Closed mode reveal that thermal stagnation produces compound losses rather than simple trade-offs, with simultaneous degradation of thermal efficiency (4.89% vs. 11.31% in Normal mode) and electrical performance (−6.69% vs. −0.64% in Normal mode) due to heat accumulation.
- Quantification of hydraulic regime dominance: Thermal energy production exhibits a variation of 693% across operating modes, while electrical generation varies by only 34%, establishing the hydraulic regime as the primary determinant of thermal performance, with secondary effects on electrical generation arising from temperature-dependent efficiency.
- Confirmation of Pareto improvement: Multi-dimensional analysis demonstrates that Forced circulation constitutes a true Pareto improvement over natural circulation (110–121% across all metrics), rather than a trade-off between competing objectives, expanding the performance frontier rather than merely moving along it.
- Significance of thermal contribution: In properly configured systems, thermal energy accounts for 43% of total energy output (compared to 13% in stagnant systems), demonstrating that PVT can deliver substantial thermal benefits beyond simply recovering waste heat associated with electrical degradation.
- Quantification of active cooling benefits: Forced circulation reduces cell operating temperature sufficiently to produce a net electrical gain despite thermal energy extraction, with the 0.70% improvement corresponding to approximately 31 Wh/day of additional generation, partially offsetting pump energy consumption.
- Criticality of generation-demand matching: The consistent temperature decrease at the end of the day across all modes indicates that the observed thermal efficiencies (4.89–13.67%) represent lower bounds imposed by the consumption profile rather than system capacity, suggesting that improved alignment between energy generation and demand could yield additional gains in practical applications.

## 5. Conclusions and Future Works

The results presented in this work have particularly relevant implications for the residential energy sector in Brazil, where electric water heating accounts for approximately 13% of total residential electricity consumption and contributes significantly to evening peak demand. The demonstrated performance of the PVT system with total energy gains of 74 to 77% compared to conventional photovoltaic systems under active hydraulic regimes suggests that the adoption of hybrid photovoltaic–thermal technology can simultaneously address two critical challenges of the Brazilian power grid: reducing overall residential consumption and alleviating peak demand stress caused by the widespread use of electric showers during evening hours.

Notably, the modular architecture of the system evaluated in this study enables a straightforward retrofit pathway: existing rooftop photovoltaic installations can be converted into PVT systems by adding heat exchanger modules mounted on the back of the photovoltaic panels, without requiring panel replacement or significant structural modifications. This retrofit approach is particularly attractive for the growing number of residential and commercial PV systems already installed across Brazil, as it allows users to harness substantial thermal energy (3.1 to 3.4 kWh/day, as demonstrated in this work) from panels that would otherwise generate only electricity.

Replacing electric shower consumption with solar-heated water not only reduces electricity costs and grid demand but also mitigates the environmental impact associated with peak-time electricity generation, which, in the Brazilian context, occasionally relies on fossil-fuel-based thermal power plants to meet seasonal demand increases.

Future work will focus on simultaneously comparing the three circulation modes under identical environmental conditions. To enable this analysis, the experimental setup will be expanded through the installation of three independent PVT systems in the same period. Another research direction involves developing a model predictive controller to optimally schedule pump operation, thereby reducing energy costs at the consumer unit. Finally, these strategies will be implemented and evaluated in a real-scale experimental home energy management system integrated with load scheduling and battery management.

## Figures and Tables

**Table 1:** Quantities monitored by the data acquisition system.

Quantity	Symbol	Unit
Module PV-1 DC voltage	$V_{PV1}$	V
Module PV-2 DC voltage	$V_{PV2}$	V
Module PVT-1 DC voltage	$V_{PVT1}$	V
Module PVT-2 DC voltage	$V_{PVT2}$	V
Module PV-1 DC current	$I_{PV1}$	A
Module PV-2 DC current	$I_{PV2}$	A
Module PVT-1 DC current	$I_{PVT1}$	A
Module PVT-2 DC current	$I_{PVT2}$	A
Solar irradiance	*G*	W/m^2^
Ambient temperature	$T_{amb}$	°C
Boiler water temperature	$T_{boiler}$	°C
Reservoir water temperature	$T_{res}$	°C
Heat exchanger inlet temperature	$T_{i}$	°C
Heat exchanger outlet temperature	$T_{o}$	°C
Mass flow rate	$\dot{m}$	L/s
Wind speed	$V_{wind}$	m/s

**Table 2:** Estimated uncertainty values for the sensors of the data acquisition system.

Sensor	Range	$u_{\mathrm{cal}}$	$u_{q}$	$u_{c}$	U(k=2)	U%
Voltage (V)	0 to 56	0.3297	$2.47\;\times \;10^{-4}$	0.3297	0.6594	1.178
Current (A)	0 to 50	0.1925	$2.20\;\times \;10^{-4}$	0.1925	0.3851	0.770
Pyranometer (W/m^2^)	0 to 1600	9.238	$7.05\times 10^{-3}$	9.238	18.477	1.155
Temperature (°C)	−10 to 85	0.0722	—	0.0722	0.1443	0.152
Mass flow (L/s)	0 to 0.2	$1.155\;\times \;10^{-3}$	$8.81\;\times \;10^{-7}$	$1.155\;\times \;10^{-3}$	$2.309\;\times \;10^{-3}$	1.155
Wind speed (m/s)	0 to 42	0.7275	$1.85\;\times \;10^{-4}$	0.7275	1.455	3.464

**Table 3:** Operating modes and valve states of the PVT system.

Regime	Valve 1	Valve 2	Valve 3	Valve 4	Valve 5
Normal circulation	ON	ON *	ON	ON	OFF
Forced circulation	ON	ON *	ON	OFF	ON
Closed circulation	ON	OFF	ON	ON	OFF

* Open when the water is being used for showering.

**Table 4:** Multi-day performance comparison of the three hydraulic operating modes.

Parameter	Normal	Closed	Forced
	(5 Days)	(6 Days)	(6 Days)
Energy Production			
$E_{\mathrm{PV}}$ [kWh]	4.179±0.331	3.554±0.291	4.423±0.745
$E_{\mathrm{PVT}}$ [kWh]	4.153±0.367	3.318±0.317	4.454±0.615
$Q_{\mathrm{th}}$ [kWh]	3.113±0.250	0.484±0.546	3.354±0.389
$E_{\mathrm{equiv}}$ [kWh]	7.267±0.518	3.801±0.484	7.808±0.728
Mean Efficiencies			
${\bar{\eta }}_{\mathrm{el},\mathrm{PV}}$ [%]	16.58±0.84	16.95±0.43	18.02±3.54
${\bar{\eta }}_{\mathrm{el},\mathrm{PVT}}$ [%]	16.43±0.63	15.64±0.66	18.15±3.10
${\bar{\eta }}_{\mathrm{th}}$ [%]	11.31±2.17	4.89±4.02	13.67±2.10
${\bar{\eta }}_{\mathrm{ov}}$ [%]	27.95±2.69	20.53±4.18	31.81±4.37
Performance Gains			
$\Delta_{\mathrm{el}}$ [%]	−0.64±1.20	−6.69±3.22	+0.70±1.5
$\Delta_{\mathrm{tot}}$ [%]	+74.04±7.10	+7.62±18.22	+76.53±14.73
Environmental Conditions & Quality			
*H* [kWh/m^2^]	6.240±0.726	5.151±0.468	6.185±0.623
$\bar{G}$ [W/m^2^]	595±110	554±50	621±78

## Data Availability

The datasets generated and analyzed during the current study are publicly available in Mendeley Data at https://data.mendeley.com/datasets/xwcyp5j9pz/2 (accessed on 15 February 2026).
